# Repeated photobiological regulation therapy alleviates inflammation in mice with experimental acute pancreatitis through ROS/NF-κB pathway

**DOI:** 10.1007/s10103-026-04868-7

**Published:** 2026-04-07

**Authors:** Fang Nie, Jiahui Qi, Shaolong Hao, Haowei Shi, Yang Hu, YuChuan Ding, Wei Han

**Affiliations:** 1 Central Laboratory, Beijing Luhe Hospital Affiliated to Capital Medical University, Beijing, China; 2 Department of General Surgery, Beijing Luhe Hospital Affiliated to Capital Medical University, Beijing, China; 3 Department of Neurosurgery, Wayne State University, Detroit, United States

**Keywords:** Pancreatitis, Photobiomodulation therapy, NF-κB, BAY 11-7082, Inflammation, ROS

## Abstract

The prevalence of acute pancreatitis (AP) is increasing, but current treatments remain inadequate. Photobiomodulation (PBM) therapy has demonstrated anti-inflammatory effects under various conditions; however, its role in AP has not been explored. Evaluating this could introduce a noninvasive treatment option for AP. This study aimed to assess PBM’s potential of PBM in reducing cerulein-induced AP inflammation and elucidate the underlying mechanisms. Animal Experiment: Mice were categorized into control, model, and experimental groups. AP was induced with cerulein injections in depilated mice, followed by seven PBM irradiation sessions in the experimental group. Blood samples collected 24 h post-injection were analyzed for TNF-α, IL-6, amylase, and lipase levels. The pancreatic tissue was histologically examined and scored. Cell Experiment: AR42J cells, grouped similarly, were subjected to cerulein exposure and PBM irradiation. An NF-κB inhibitor has been introduced into some groups. Measurements included amylase, NF-κB p65, phosphorylated NF-κB p65, and (Robot Operating System) ROS levels in the supernatant. Animal Experiment: PBM treatment reduced pancreatic tissue edema and serum amylase, lipase, TNF-α, and IL-6 levels. Cell Experiment: ROS levels increased, NF-κB p65 expression decreased, and TNF-α, IL-6, amylase, and lipase in the cell supernatant decreased post-PBM treatment. The addition of the NF-κB inhibitor BAY 11-7082 to the PBM-treated group did not increase ROS levels. PBM therapy shows potential as a noninvasive AP treatment by modulating ROS-mediated NF-κB signaling. Clinical studies are needed to confirm the efficacy and explore the applications of AP treatment.

**Supplementary Information:**

The online version contains supplementary material available at 10.1007/s10103-026-04868-7.

## Introduction

Acute pancreatitis (AP) is an inflammatory condition of the pancreas, which can lead to severe systemic complications [1]. AP commonly presents as an acute abdominal emergency, with clinical manifestations ranging from mild interstitial edema to extensive pancreatic parenchymal necrosis, often complicated by multiple organ dysfunction [1]. The mortality rate for patients with infected pancreatic necrosis has been reported to reach approximately 15% [2]. Despite substantial advances in our understanding of AP pathophysiology over the past two decades, there is still no approved specific targeted therapy for this disease [3].

The pathogenesis of AP is recognized as a multistep inflammatory cascade. The initial localized pancreatic inflammation is progressively amplified by a variety of inflammatory mediators, including proinflammatory cytokines, reactive oxygen species (ROS), chemokines, leukocyte adhesion molecules, lipid mediators, and gaseous messengers, ultimately driving uncontrolled pancreatic inflammatory injury [4]. Elucidating the molecular mechanisms underlying pancreatic injury and its crosstalk with the systemic inflammatory system is critical for identifying promising therapeutic targets for AP.

The research and clinical application of laser-based therapy can be traced back to the late 1960 s [5]. Since then, low-level laser therapy (LLLT) has been widely explored to regulate physiological functions in nearly all organ systems of humans and animals [6, 7]. Over the past decade, photobiomodulation (PBM) has become the standardized terminology in the field, replacing the previously used LLLT, as it more accurately encompasses the bidirectional regulatory effects (both stimulatory and inhibitory) of low-power light irradiation on biological processes. PBM is defined as a non-thermal phototherapy modality that does not induce a significant temperature rise in irradiated tissues [8]. This non-thermal process triggers photophysical and photochemical reactions via endogenous chromophores, exerting multiple therapeutic effects including enhanced tissue regeneration, accelerated wound healing, and alleviation of inflammation and pain [9–15].

Accumulating evidence from recent studies has demonstrated that PBM exerts prominent anti-inflammatory effects in various disease models, including oral inflammatory lesions, neuropathic pain, and cutaneous inflammatory disorders [16–20]. Our research group has previously characterized the biphasic dose-response relationship of the anti-inflammatory effects of PBM [17]. The present study was inspired by several pivotal preclinical findings in the field, most notably the discovery that transcranial PBM can penetrate the intact skull and exert intracranial therapeutic effects [21, 22]. This ability of PBM to treat deep-seated pathological conditions has emerged as a landmark finding, which prompted us to pose a critical scientific question: if PBM can penetrate the skull to exert biological effects, it is theoretically feasible for PBM to penetrate the abdominal wall and gastric tissue of mice to target the pancreas. Compelling evidence from previous studies has also confirmed that pre-irradiation with low-level laser (the core modality of PBM) prior to transplantation can improve the in vitro function of isolated pancreatic islets and enhance the efficacy of islet transplantation [22]. Previous studies have confirmed that the maximum penetration depth of an 810 nm laser can extend up to 10 mm [23, 24], while the abdominal skin of mice is extremely thin. Consequently, the 810 nm laser is capable of penetrating the abdominal wall and reaching the abdominal cavity in mice. Building on this prior evidence, we hypothesized that PBM could exert a therapeutic effect on AP. Accordingly, the primary objectives of this study were to investigate the therapeutic efficacy of PBM in a caerulein-induced mouse model of AP, and to elucidate the molecular mechanisms underlying its protective effects.

While PBM has been well documented to alleviate pain and inflammation and promote tissue regeneration, its therapeutic effect and underlying mechanisms in AP remain largely unelucidated. Unlike superficial inflammatory lesions, the pancreas is a deep abdominal organ, which poses a challenge for direct laser irradiation. However, recent studies have indicated that PBM can still exert protective and regulatory effects on deep visceral tissues via transcutaneous irradiation, as validated in aging animal models [25]. Therefore, this study proposes that PBM may exert anti-inflammatory effects on AP via transcutaneous abdominal irradiation near the pancreatic inflammatory lesion.

## Materials and methods

### Main reagents and instruments

Caerulein and BAY 11–7082 were obtained from MCE (Shanghai, China). BAY 11–7082 is a widely recognized NF-κB inhibitor. We detected total NF-κB p65 using a rabbit polyclonal antibody (ProteinTech, Cat. No. 10745-1-AP) and phosphorylated NF-κB p65 (Ser536) using a rabbit monoclonal antibody (Cell Signaling Technology, #3031). β-Actin was employed as a loading control, with the corresponding antibody purchased from Cell Signaling Technology (Beverly, MA, USA). Secondary anti-rabbit and anti-mouse IgG antibodies were also sourced from Cell Signaling Technology. Reactive oxygen species (ROS) were quantified using a detection kit from Solarbio (Beijing, China). Amylase and lipase activities were measured using assay kits provided by Nanjing Jiancheng Bioengineering Institute (Nanjing, China). Finally, the concentrations of TNF-α and IL-6 in serum samples were determined using ELISA kits from Enzyme-linked Biotechnology (Shanghai, China).

The 810 nm PBM therapy device used in this study was the RLT-24 animal laser therapy instrument (Ruiduo Life Science and Technology Co., Ltd., Shenzhen, China). An 810 nm wavelength was selected for its near-infrared properties, providing minimal thermal effect, deep tissue penetration, and minimal absorption by tissue media [26]. Other key instruments included a Cytation 5 multifunctional microplate reader (Agilent Biotek, USA), a Leica CM1950 cryostat (Leica Microsystems, Germany), and a protein electrophoresis and wet transfer system (Bio-Rad Laboratories, Inc., USA).

### Experimental animals and ethical statement

Specific pathogen-free (SPF) male C57BL/6J mice aged 6–8 weeks were purchased from Beijing Vital River Laboratory Animal Technology Co., Ltd. Mice were housed in a SPF barrier environment with constant temperature (24 ± 1 °C), constant humidity (50% ± 5%), and a 12 h light/dark cycle, with ad libitum access to sterile food and water [27].

All animal procedures in this study strictly complied with the ARRIVE guidelines, and were reviewed and approved by the Laboratory Animal Ethics Committee of Capital Medical University (Ethics Approval No. AEEI-2025-177). All operations were performed in accordance with the 3R principles (Replacement, Reduction, Refinement) and standard animal ethical norms.

### In vivo animal experiments

#### Establishment of AP model

We employed caerulein alone to induce AP in mice, as this method specifically models edematous (mild to moderate) pancreatitis, closely mimicking the early lesions of human AP. The severity of AP was precisely controlled by adjusting the number of caerulein injections (6–10 times). This approach is supported by previous studies demonstrating its dose-dependent relationship and reproducibility [28]. All mice received a fixed dose of caerulein (50 µg/kg) via intraperitoneal injection at consistent intervals. Model establishment was confirmed through multiple endpoints, including serum amylase/lipase detection, pancreatic edema index, and histopathological scoring [29]. Animals were randomly grouped, and all histopathological evaluations were performed blindly to reduce bias [30].

To induce pancreatitis, age- and sex-matched C57BL/6J mice were fasted for 18 h, but provided with water ad libitum (*n* = 6 per experimental group). Mice received seven hourly intraperitoneal injections of either saline (control) or cerulein (50 µg/kg/h in saline). Mice were euthanized by CO₂ asphyxiation 1 h after the final caerulein injection, and blood and tissue samples were collected. The severity of cerulein-induced pancreatitis was assessed using H&E-stained tissue sections graded by two independent, blinded observers, as previously described [31].

#### Experimental grouping and PBM intervention protocol

**Fig. 1:**
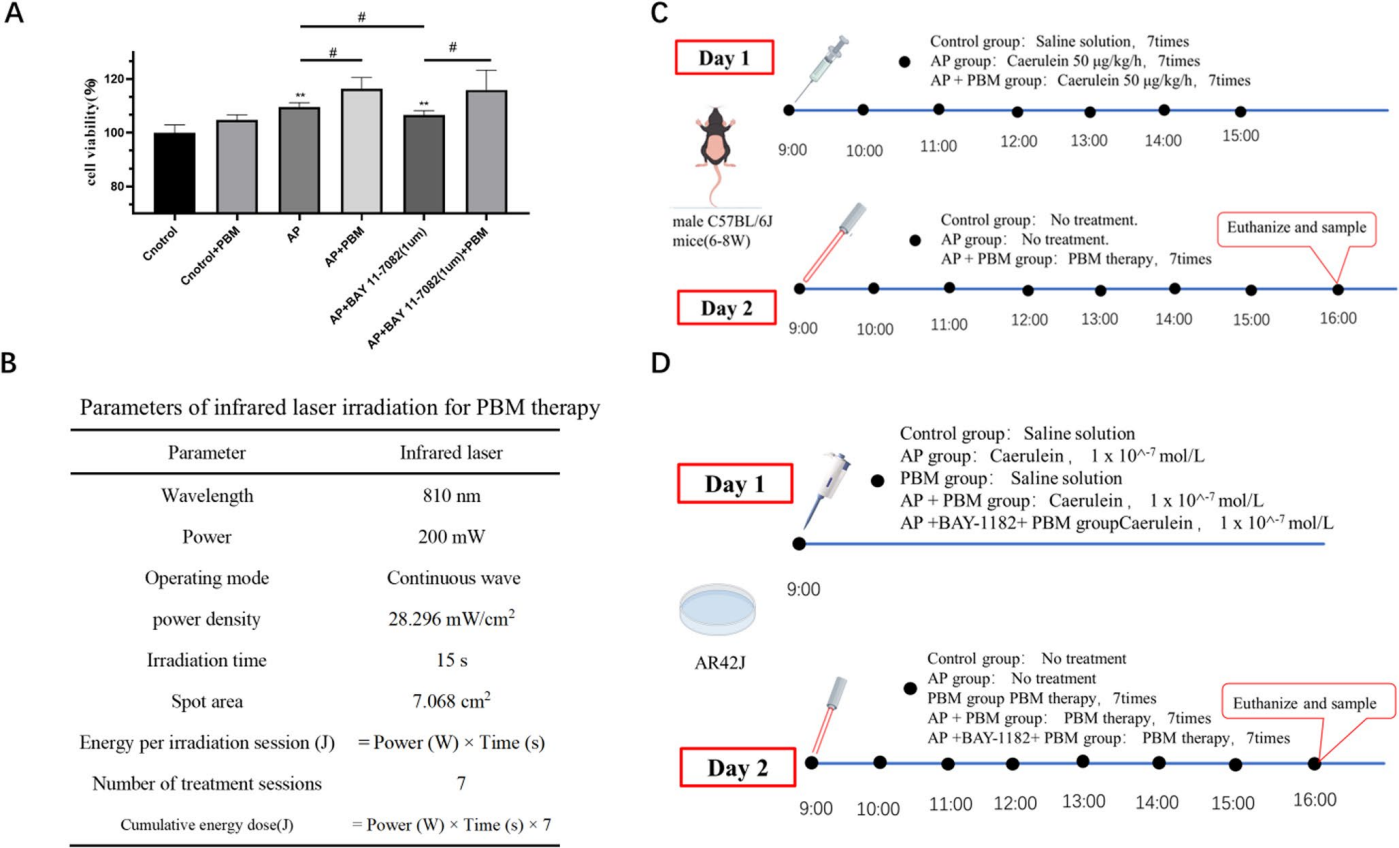
Comprehensive Overview of PBM Treatment: Efficacy, Parameters, and Experimental Protocols. (**A**) Effects of PBM on AR42J Cell Viability (CCK-8 Assay).PBM irradiation with Panel B parameters did not compromise AR42J cell proliferation. (**B**) Specification of PBM Parameters. Critical PBM parameters for in vivo/in vitro experiments: wavelength, power density, irradiation duration. Parameters in Panels C & D derived from this section. (**C**) Experimental Timeline for In Vitro Studies. Acute pancreatitis (AP) group: Day 1: AP model induction via 7× hourly intraperitoneal caerulein injections (50 µg/kg).(Commencing 9:00 AM) Day 2: 7× hourly PBM irradiations (Starting 9:00 AM) (**D**) Experimental Timeline for In Vivo Studies AR42J cell experiments: Grouping and timeline structure identical to Panel C. Abbreviations: *PBM* photobiomodulation, *AP* acute pancreatitis, *NF-κB* nuclear factor kappa-B, *p-NF-κB* phosphorylated nuclear factor kappa-B

The experimental grouping and intervention timeline are detailed in Fig. 1C, and the key parameters of PBM irradiation are summarized in Fig. 1B. PBM intervention was initiated on the day following the final caerulein administration, consisting of 7 consecutive irradiations delivered at 1-hour intervals.

To ensure unobstructed exposure of the target abdominal irradiation field, mice underwent abdominal depilation 2 days prior to irradiation. During the procedure, the laser probe was positioned vertically 1 cm above the mouse abdomen, and non-target regions were fully shielded with aluminum foil to prevent off-target exposure. Fixed-point abdominal irradiation was performed using an 810 nm near-infrared laser, with a 650 nm visible light as the alignment beam to precisely calibrate the irradiation area and maintain a consistent laser spot size of 3 cm². Identical irradiation parameters were used for both in vivo and in vitro experiments.

#### Anesthesia and euthanasia

Mice were anesthetized using 2% isoflurane in oxygen (flow rate 1 L/min). Anesthesia depth was confirmed by the absence of hind paw withdrawal reflex to noxious pinch, with monitoring limited to pre-procedure assessment due to the brief duration (< 5 min) of each experimental intervention.

At the experimental endpoint, mice were euthanized by gradual CO₂ asphyxiation under maintained deep anesthesia, followed by cervical dislocation to ensure death. All animal handling and experimental procedures were carried out in strict accordance with the Guide for the Care and Use of Laboratory Animals published by the United States National Research Council (NRC), as well as the pre-approved animal experimental ethics protocol.

#### Biological sample collection

Before euthanizing the mice, terminal whole blood samples were collected from each group via retro - orbital sinus puncture, with the mice kept under deep anesthesia throughout the procedure. The freshly obtained blood samples were promptly centrifuged at 12,000 × g for 10 min at 4 °C to separate out cellular debris. Subsequently, the upper serum layer was carefully transferred, divided into aliquots, and stored at − 80 °C until further biochemical and immunoassays were conducted.

Right after blood collection and euthanasia, the pancreatic tissues of the mice were swiftly harvested on an ice - cooled surface. Excess adipose and connective tissues were trimmed away, and the tissues were then split into two equivalent portions. One portion was fixed in a 4% (w/v) paraformaldehyde (PFA) solution to prepare for subsequent paraffin embedding and histological examination. The other portion was immediately flash - frozen in liquid nitrogen to maintain protein integrity and subsequently stored at − 80 °C for future Western blotting and other protein - detection assays.

#### Histology & scoring

After harvesting, the pancreatic tissues were immediately fixed in 4% paraformaldehyde (PFA) to preserve tissue architecture. Following fixation, a graded ethanol dehydration series (70%, 85%, 95%, 100%) was performed for 15–30 min per step to remove residual water. The dehydrated tissues were then embedded in Optimal Cutting Temperature (OCT) compound and rapidly frozen at −17 °C. Cryosections (6 ± 0.5 μm) were prepared using a Leica CM1950 cryostat (Leica, Germany). Sections were stained with Hematoxylin and Eosin (H&E) for histological evaluation. Pancreatic tissue sections were examined in a blinded manner. Histological scores were assigned based on the established criteria, evaluating the extent and severity of edema, inflammatory cell infiltration, and acinar necrosis. Each parameter was graded on a 0–5 scale. The final scores for each specimen were calculated as the average of the scores from two independent investigators. The degree of inflammation and edema was assessed using a semi-quantitative scoring system (S.C.S.), ensuring unbiased evaluation across treatment groups [32].

#### Enzyme-linked immunosorbent assay

Serum samples from mice in each in vivo experimental group were analyzed via ELISA to quantify serum IL-6 and TNF-α levels, strictly following the manufacturer’s protocol.

### In vitro cell experiments

#### Cell culture

The AR42J rat pancreatic acinar cell line, which has been well characterized in previous studies [33, 34], was obtained from Shanghai Zhongqiao Xinzhou Biotechnology Co., Ltd. (Catalog No. ZQ0145). Cell line identity was authenticated via short tandem repeat (STR) genotyping, and all cell batches were verified to be free of mycoplasma contamination using a luminescence-based assay before formal experiments. Cells were maintained in complete culture medium consisting of high-glucose Dulbecco’s Modified Eagle Medium (DMEM) supplemented with 10% (v/v) fetal bovine serum (FBS), 100 U/mL penicillin, and 100 µg/mL streptomycin. Cells were incubated in a humidified atmosphere at 37 °C with 5% CO₂, and routine subculture was performed when cells reached 80%–90% confluence. For laser irradiation experiments, cells were seeded in opaque black-walled 96-well plates to prevent optical crosstalk between adjacent wells, with each cell-seeded well bordered by media-only blank wells to further eliminate stray light interference. Plates assigned to the non-irradiated control groups were completely wrapped in aluminum foil to block all ambient and stray laser exposure throughout the irradiation process.

#### Cell model establishment and grouped intervention

An in vitro model of AP was established by treating AR42J rat pancreatic acinar cells with 1 × 10⁻⁷ mol/L caerulein for 24 h, as previously described. The experiment comprised six groups with the following interventions:

- Blank control group: Cells were cultured under standard conditions without caerulein stimulation, drug treatment, or laser irradiation.
- AP model group: Cells were stimulated with 1 × 10⁻⁷ mol/L caerulein for 24 h without further intervention.
- AP + Inhibitor group: Cells were simultaneously treated with 1 µmol/L BAY 11–7082 (a specific NF-κB pathway inhibitor) and caerulein throughout the 24-h stimulation period.
- PBM-only group: Cells underwent 7 consecutive photobiomodulation (PBM) irradiations at 1-h intervals, without caerulein stimulation or drug treatment.
- AP + PBM group: Following the initiation of caerulein-induced AP modeling, cells received 7 consecutive PBM irradiations at 1-h intervals.
- AP + Inhibitor + PBM group: Cells were treated with BAY 11–7082 throughout the 24-h caerulein stimulation period while simultaneously receiving 7 doses of PBM irradiation at 1-h intervals.
- The detailed experimental workflow is depicted in Fig. 1D, and the core PBM parameters are outlined in Fig. 1B. All PBM irradiation settings were identical to those used in the in vivo experiments.

#### CCK-8 cell viability assay

The Cell Counting Kit-8 (CCK-8, Beyotime, China) assay is based on the principle that the water-soluble tetrazolium salt WST-8 (2-methoxy-5-ethyl-3-indolyl-2-sulfonate sodium) is reduced by cellular dehydrogenases in metabolically active cells to generate an orange-colored formazan product. This reaction does not require cell lysis, and the resulting formazan is highly water-soluble. The intensity of the orange color, measured as absorbance at 450 nm (OD450), is directly proportional to the number of viable cells present in the sample.

#### Determination of intracellular ROS

To mitigate laser scattering interference, AR42J cells were seeded in black 96-well plates before experimental treatment. The experimental grouping referred to Fig. 1D, and the PBM parameters were based on Fig. 1B.

During PBM irradiation, non-irradiated control groups were fully shielded with aluminum foil to eliminate optical crosstalk between samples. The reactive oxygen species (ROS) detection protocol comprised the following steps:

- Step 1: Reagent Addition
  - After the final PBM cycle, all groups (including irradiated and control) were immediately transferred to a standard cell culture incubator for a 30-min reincubation period to allow cellular recovery.
  - Subsequently, ROS detection reagent was uniformly added to all wells across groups to ensure consistent assay conditions.
- Step 2: Fluorescence Quantification

Fluorescence signals were quantified using a BioTek Cytation^®^ 5 multimode microplate reader (Agilent Technologies, Winooski, VT, USA) in strict accordance with the manufacturer’s protocol.

#### Protein extraction and western blotting

Protein extraction was performed using RIPA lysis buffer supplemented with protease and phosphatase inhibitors from AR42J cells and mouse pancreatic tissue. The cell lysate was centrifuged at 12,000 rpm for 10 min; tissue samples were ground into a homogenate in liquid nitrogen, followed by sonication and centrifugation.

The supernatant was mixed with 5× SDS loading buffer, denatured at 100℃ for 8 min, and then separated by SDS-PAGE. After transferring the proteins onto a PVDF membrane, the membrane was incubated overnight at 4℃ with primary antibodies (β-actin 1:10,000; Anti-NF-κB p65 1:5,000; anti-phospho-NF-κB p65 1:2,000).

### Determination of amylase and lipase

In Vivo Animal Experiments. Blood samples were collected from mice in each group via retro-orbital sinus puncture. Serum amylase and lipase levels were quantified as follows (Fig. 1C): whole blood was immediately centrifuged at 12,000 × g for 10 min at 4 °C. The supernatant serum was then separated and stored at −80 °C pending analysis.

In Vitro Cell Experiments. Culture supernatants from each experimental group (Fig. 1D) were harvested for amylase quantification.

Quantitative Analysis. For both experimental models, the target enzymes were assayed using commercial kits (Bio Assay Systems, Hayward, CA, USA): In vivo: Serum amylase and lipase. In vitro: Culture supernatant amylase.

All procedures strictly adhered to the manufacturer’s protocols.

### Statistical analysis

GraphPad Prism software (version 7.0) (GraphPad Software, San Diego, CA, USA, www.graphpad.com) was used for statistical analysis. At least six animals were included in each group for animal experiments to meet statistical requirements, and in vitro studies were repeated at least thrice in each group. The evaluator was blinded to the animal or drug treatment conditions. A two- tailed Student’s t-test was employed for comparisons between two experimental groups. Differences in means across multiple groups were analyzed using one-way analysis of variance (ANOVA) followed by Tukey’s post hoc analysis. Data are presented as mean ±standard error of the mean (SEM).

## Results

### Effect of PBM on caerulein-induced acute pancreatitis and its preliminary mechanism

Establishment of the PBM Irradiation Protocol and Verification of Its Biosafety in Pancreatic Acinar Cells. To devise a standardized PBM protocol for the treatment of AP, we implemented a consistent 810 nm PBM irradiation strategy across both in vivo and in vitro experiments. This strategy featured a fixed power density of 28.296 mW/cm² and a single irradiation duration of 15 s (as detailed in Fig. 1B). Initially, we assessed the biosafety of this protocol in AR42J rat pancreatic acinar cells using the CCK-8 assay. The findings indicated that PBM irradiation with the specified parameters did not hinder the proliferation of AR42J cells, with no statistically significant difference in cell viability observed between the PBM-only group and the blank control group (*p* > 0.05, Fig. 1A). This confirmed the non-cytotoxic nature of the established PBM protocol.

Protective Impact of PBM on Caerulein-Induced Acute Pancreatitis in Mice. To substantiate the therapeutic efficacy of PBM on AP in vivo, we induced a mouse AP model through seven hourly intraperitoneal injections of caerulein (50 µg/kg body weight), commencing the first injection at 9:00 on Day 1. The experimental grouping and treatment schedule are illustrated in Fig. 1C: on Day 2, mice in the PBM treatment groups underwent seven rounds of hourly PBM irradiation starting at 9:00, utilizing the same parameters outlined in Fig. 1B. At 16:00 on Day 2, retro-orbital venous blood and pancreatic tissues were collected for subsequent analysis. Histological examination revealed that PBM treatment significantly alleviated pancreatic edema, inflammatory cell infiltration, and acinar cell necrosis in AP mice. Concurrently, serum amylase levels and pro-inflammatory cytokine levels in the PBM treatment group were notably lower than those in the untreated AP group (*p* < 0.05).

In Vitro Anti-Inflammatory Effects of PBM on Caerulein-Stimulated AR42J Cells and Its Preliminary Mechanism. To delve deeper into the protective mechanism of PBM on AP, we conducted in vitro experiments using AR42J cells, establishing an inflammatory model through 24-hour stimulation with 1 × 10⁻⁷ mol/L caerulein. The grouping and treatment timeline mirrored those of the in vivo experiment (Fig. 1D), with cells categorized into six groups receiving parallel treatments: (1) Control group, with no intervention; (2) AP group, treated with 1 × 10⁻⁷ mol/L caerulein for 24 h; (3) AP + inhibitor group, co-treated with 1 × 10⁻⁷ mol/L caerulein and 1 µmol/L NF-κB pathway inhibitor BAY 11–7082; (4) PBM-only group, subjected to seven rounds of hourly PBM irradiation without caerulein stimulation; (5) AP + PBM group, receiving seven rounds of hourly PBM irradiation following the establishment of the caerulein-induced AP model; (6) AP + inhibitor + PBM group, undergoing BAY 11–7082 intervention and PBM irradiation after caerulein stimulation. All PBM treatments adhered to the parameters specified in Fig. 1B. The results demonstrated that PBM treatment significantly reduced the expression of pro-inflammatory factors in caerulein-stimulated AR42J cells, with this protective effect aligning with the intervention effect of the NF-κB inhibitor BAY 11–7082. This suggests that the anti-inflammatory effect of PBM may be mediated through the regulation of the NF-κB signaling pathway.

### Therapeutic Effects of PBM on Caerulein-Induced Acute Pancreatitis in Mice

**Fig. 2:**
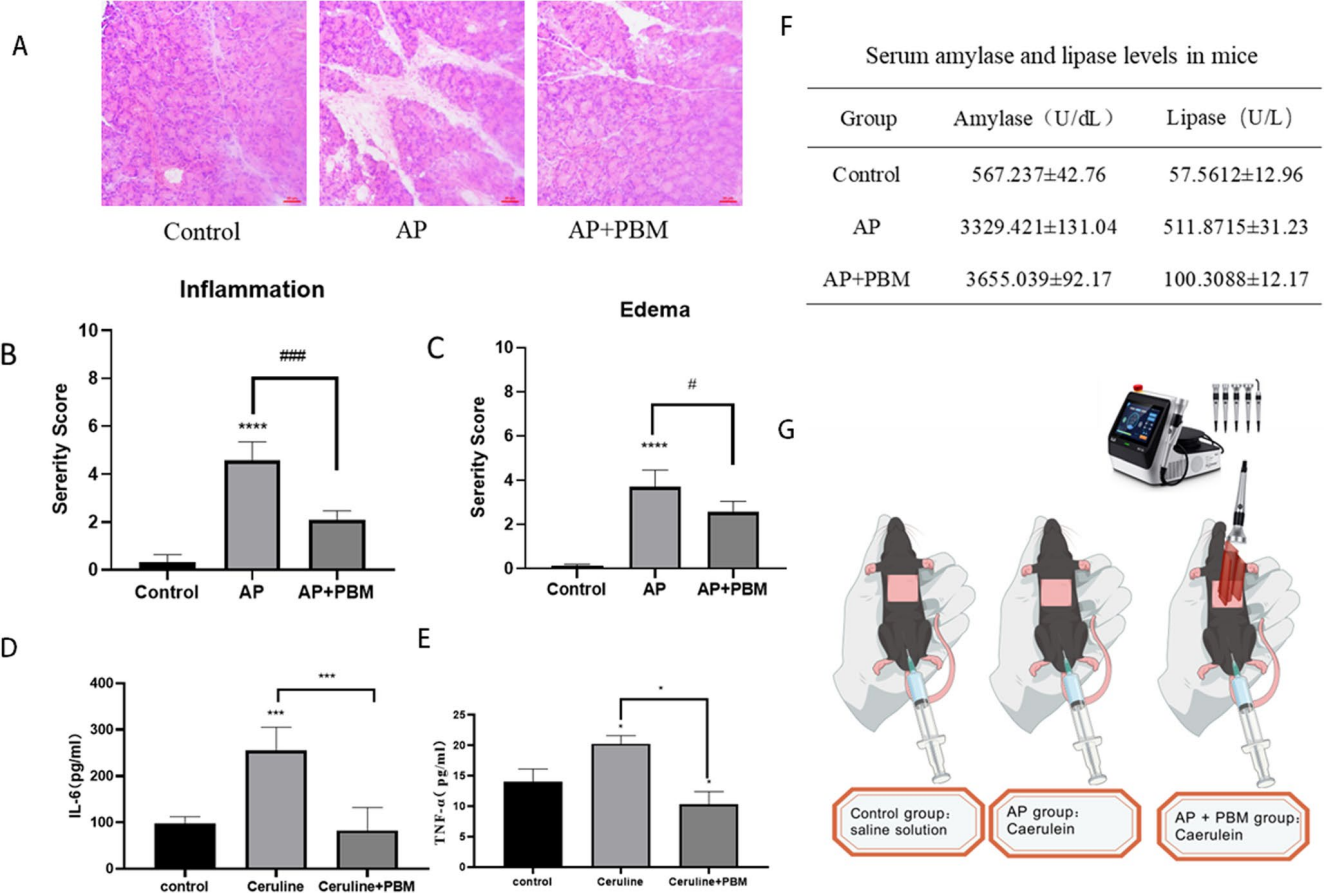
Therapeutic effects of PBM in mice with caerulein-induced AP.(**A**) Representative hematoxylin-eosin (HE) staining images of pancreatic tissues harvested at 24 h after the final caerulein injection (magnification ×200).(B-C) Quantitative pathological scores for inflammatory infiltration (**B**) and edema (**C**) based on the HE staining results in panel A.(**D**-**E**) Serum levels of interleukin-6 (IL-6, **D**) and tumor necrosis factor-α (TNF-α, **E**) detected by enzyme-linked immunosorbent assay (ELISA).(**F**) Serum levels of pancreatic injury markers amylase and lipase in each experimental group.(**G**) Schematic diagram of the in vivo PBM irradiation setup. The colored area on the mouse abdomen indicates the depilated irradiation region; the 810 nm laser used for formal PBM treatment was invisible to the naked eye, and a 810 nm visible laser was used as the alignment beam. *P* < 0.05, *P < 0.005, *P* < 0.0001 vs. the blank control group; #*P* < 0.05, ##*P* < 0.005, ###*P* < 0.001 vs. the untreated AP group. Error bars represent standard deviation (SD)

Figure 2 revealed that PBM effectively mitigated inflammatory infiltration and edema in a mouse model of caerulein-induced AP. Specifically, Fig. 2A displayed hematoxylin-eosin (HE)-stained images of pancreatic tissues collected at 24 h after the final caerulein injection, which confirmed the successful establishment of the AP model in the caerulein-treated group and demonstrated that PBM treatment significantly alleviated pancreatic inflammatory damage. Quantitative analysis of the pathological scores for inflammatory infiltration (Fig. 2B) and edema (Fig. 2C) further showed that PBM substantially reduced the severity of pancreatic edema and inflammatory infiltration in AP model mice (*p* < 0.05 compared with the untreated AP group).

At the same time point of 24 h after the final caerulein injection, retro-orbital venous blood samples were collected from mice, and the serum levels of pro-inflammatory cytokines interleukin-6 (IL-6) and tumor necrosis factor-α (TNF-α) were measured by ELISA. As shown in Fig. 2D and E, caerulein stimulation induced a marked elevation of serum IL-6 and TNF-α levels in AP model mice compared with the blank control group (*p* < 0.05), while PBM treatment significantly reversed this increase (*p* < 0.05 vs. the untreated AP group), indicating that PBM exerted a pronounced anti-inflammatory effect in vivo.

We further evaluated the effect of PBM on the serum levels of pancreatic injury markers amylase and lipase, with the results presented in Fig. 2F. Compared with the blank control group, serum amylase and lipase levels were significantly increased in the AP model group (*p* < 0.05), which were notably reduced by PBM treatment (*p* < 0.05 vs. the untreated AP group), further confirming the protective effect of PBM against pancreatic injury in AP mice.

### Therapeutic effects of PBM on acute pancreatitis in cell

**Fig. 3:**
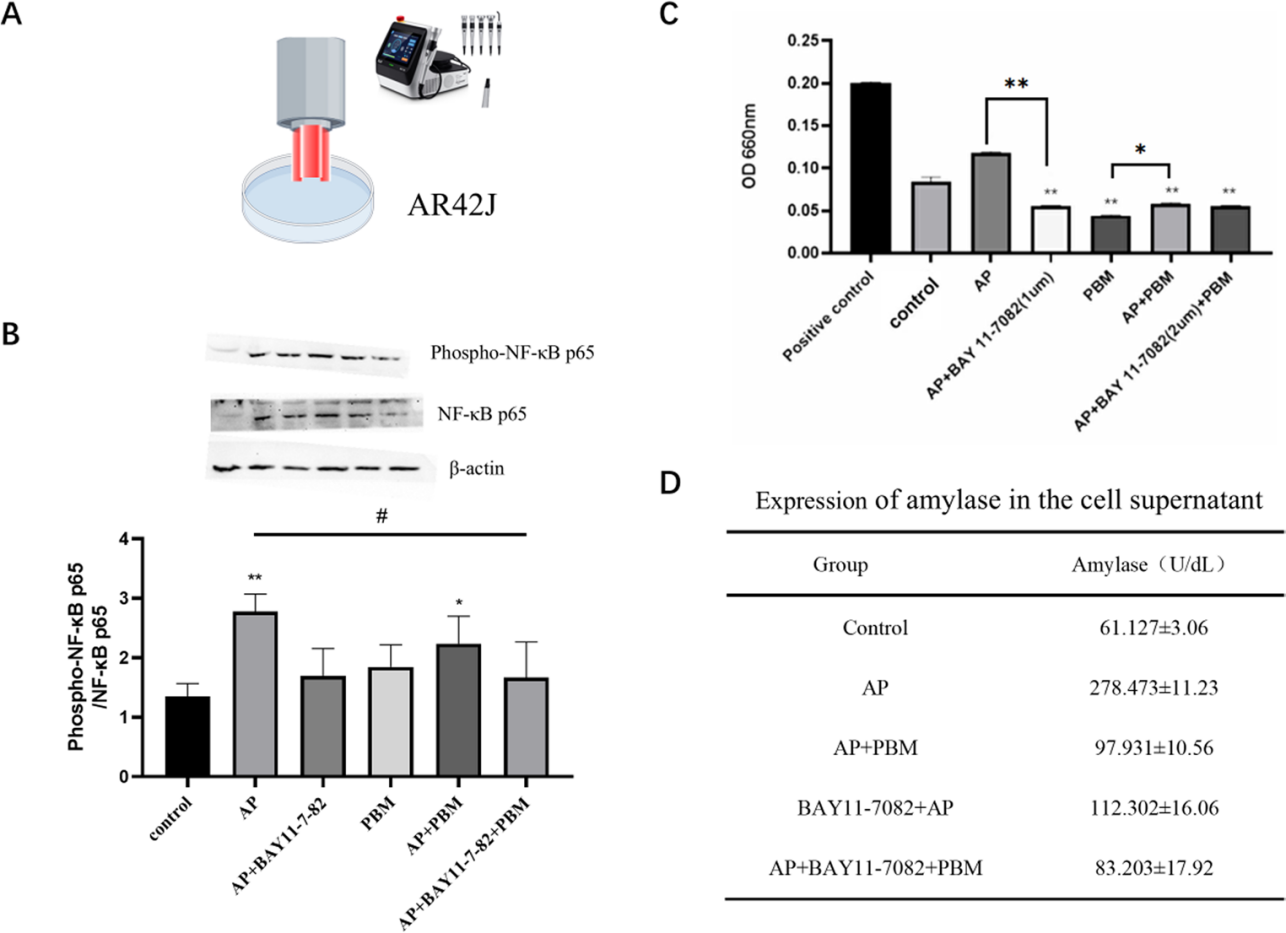
PBM exerts a protective effect on in vitro AP via regulating the NF-κB signaling pathway, with a mechanistic convergence to NF-κB inhibitor BAY 11–7082(**A**) Schematic diagram of the experimental workflow for PBM and BAY 11–7082 intervention in the arachidonic acid-induced AR42J cell AP model.(**B**) Immunoblotting analysis and semi-quantification of total NF-κB and p-NF-κB protein expression in AR42J cells of each experimental group.(**C**) Intracellular ROS levels in each group, detected using a ROS fluorescent probe at 0.5 h after the final PBM irradiation.(**D**) Quantitative detection of α-amylase levels in the culture supernatant of AR42J cells in each group. *P* < 0.05, *P < 0.01, *P* < 0.0001 vs. the blank control group; #*P* < 0.05, ##*P* < 0.01, ###*P* < 0.001 vs. the untreated AP group. Error bars represent standard deviation (SD)

Figure 3D summarizes the therapeutic effect of photobiomodulation (PBM) in the in vitro model of AP. Key findings showed that α-amylase concentration was significantly elevated in the AP group compared with the blank control group (*P* < 0.05), which verified the successful establishment of the cell AP model. Compared with untreated AP samples, PBM intervention significantly reduced α-amylase levels (*P* < 0.05), confirming the therapeutic effect of PBM in this model. After pharmacological inhibition with BAY 11–7082, a specific NF-κB pathway inhibitor, α-amylase levels were partially decreased compared with the AP control group, but remained higher than the baseline level of the blank control group. No significant difference in α-amylase levels was observed between the BAY 11–7082 monotherapy group and the BAY 11–7082/PBM combination group (*P* > 0.05). These data indicated a mechanistic convergence between PBM and BAY 11–7082 via the NF-κB signaling pathway. No additive effect was detected in the combination treatment group, suggesting that the therapeutic effect of PBM under these experimental conditions is mainly mediated by regulating the NF-κB pathway.

The results of ROS detection are shown in Fig. 3C. ROS measurement was performed using a commercial ROS detection kit at 0.5 h after the final laser irradiation. The results revealed that ROS levels were significantly reduced in the PBM-treated group, while remaining high in the AP group. This finding suggested that PBM may exert its therapeutic effect by regulating ROS production. Interestingly, we also found that BAY 11–7082 effectively downregulated ROS levels, which further supported the inhibitory effect of this agent on the inflammatory cascade.

To explore the inhibitory mechanism of PBM on arachidonic acid-induced AP, we examined the expression levels of NF-κB and its phosphorylated form (p-NF-κB) in pancreatic acinar cells. These proteins are critical for the regulation of inflammatory responses, and their expression may be modulated by ROS [32]. The experimental results are presented in Fig. 3B: compared with the blank control group, the proportion of phosphorylated NF-κB was significantly increased in the AP group (*P* < 0.05), which further validated the successful establishment of the AP model. The level of phosphorylated NF-κB in the AP + PBM treatment group was still higher than that in the blank control group. There was no significant difference in the proportion of phosphorylated NF-κB between the AP + BAY 11–7082 inhibitor group and the blank control group (*P* > 0.05), indicating that the inhibitor exerted the expected blocking effect on NF-κB activation. The phosphorylation level of NF-κB in the AP + BAY 11–7082 + PBM group was significantly lower than that in the AP group (*P* < 0.05). Figure 3A shows the schematic diagram of PBM intervention in the AR42J cell AP model.

## Discussion

The global incidence of acute pancreatitis (AP) is continuously rising, exceeding 3% worldwide [35]. The global incidence of acute pancreatitis (AP) is continuously rising, exceeding 3% worldwide.

Key pathogenic cellular events in AP include abnormal calcium signaling, mitochondrial dysfunction, and endoplasmic reticulum stress [36–38]. AP onset is triggered by acinar cell toxins (e.g., alcohol, cholecystokinin hyperstimulation, nicotine, bile acids) [38, 39],, which induce zymogen granule-lysosome fusion, trypsinogen activation, and cellular apoptosis [1]. Additionally, infiltrating macrophages engulf trypsinogen, convert it to trypsin, and exacerbate local acute inflammation [40]. AP is characterized by acinar cell pathology and robust inflammatory responses [41], with oxidative stress closely linked to disease severity across models and clinical settings [42]. Oxidative stress activates inflammatory cascades via macrophages and neutrophils, driving pro-inflammatory cytokine (IL-6, IL-1β, TNF-α) production [43].

NF-κB is pivotal in regulating inflammation, proliferation, differentiation, and apoptosis, with its activity context-dependently modulated (enhanced or suppressed) by reactive oxygen species (ROS) [44]. As a key downstream target of ROS in inflammation, cytotoxicity, and cancer progression [45, 46],, ROS specifically activates NF-κB signaling—supporting our hypothesis that ROS/NF-κB pathway hyperactivation contributes to AP pathogenesis [46].

PBM exerts beneficial effects on cells/tissues, with its mechanisms partially elucidated by recent advances: photons absorbed by tissue chromophores stimulate mitochondria, depolarize mitochondrial membrane potential (MMP), and increase ATP, cAMP, and ROS levels [47–49]. Given the tight link between ROS and inflammation [50, 51], we hypothesized PBM alleviates AP inflammation via ROS modulation.

While PBM’s anti-inflammatory effects in superficial inflammation are well-documented, its role in internal inflammation remains understudied. This study evaluated PBM’s efficacy in AP internal inflammation and its ROS-related mechanism. In our animal model, PBM significantly reduced inflammatory responses (evidenced by histopathological scores and serum amylase levels) compared to controls. Cell experiments further confirmed PBM inhibits NF-κB phosphorylation via ROS modulation, reducing NF-κB-regulated downstream inflammatory cytokines (TNF-α, IL-6) and mitigating inflammation.

PBM modulates ROS in various inflammatory diseases, typically via direct irradiation; recent studies suggest its therapeutic effects may extend to non-irradiated adjacent sites (e.g., brain degenerative diseases) [52] —a hypothesis supported by our findings, expanding PBM’s potential from superficial to internal diseases.

This study addresses the above key issues with three major innovative advances:

1. First in vivo validation of transcutaneous PBM for acute pancreatitis. To our knowledge, this is the first study to systematically investigate transcutaneous PBM in a cerulein-induced mouse model of AP. We show that external light irradiation significantly reduces serum amylase (a key marker of pancreatic injury) and improves histopathological scores for pancreatic edema and inflammation, without direct surgical exposure of the pancreas. These results confirm that PBM can penetrate deep tissues and exert anti-inflammatory effects on visceral organs such as the pancreas.
2. Elucidation of the molecular mechanism of PBM in AP. We clarify the protective pathway of PBM: it induces a moderate mitochondrial ROS burst, which in turn inhibits NF-κB phosphorylation and the release of pro-inflammatory cytokines (TNF-α, IL-6). These findings provide a new theoretical basis for PBM in visceral inflammation, supporting its action through ROS-mediated hormesis rather than direct ROS scavenging, and deepen the mechanistic understanding of PBM.
3. High clinical translational potential. Given the lack of specific pharmacotherapies for AP, our results suggest that transcutaneous PBM represents a safe, non-invasive, low-cost, and easily accessible adjuvant therapy. This work fills an unmet clinical need, establishes a solid experimental basis for future clinical trials, and promotes the translational application of PBM from preclinical research to clinical AP management.

In conclusion, our study provides promising evidence for the effectiveness of PBM therapy in reducing inflammation and tissue damage in AP. To reiterate, our research offers compelling support for the efficacy of PBM in mitigating inflammation and tissue injury associated with AP. Looking ahead, PBM holds potential for further development as a clinical treatment for acute pancreatitis.

## Conclusion

Our findings demonstrate that PBM, as an independent therapy, can alleviate cerulein-induced AP by reducing inflammation and the expression of biochemical markers associated with the inflammatory response. Additionally, our cell model experiments confirmed that PBM exerts these anti-inflammatory effects through the ROS/NF-κB signaling pathway.

The interesting findings of the animal experiments in this study demonstrated that PBM can function even if it cannot irradiate the lesion, which may be related to the ROS produced by PBM. The remarkable anti-inflammatory capacity observed in vivo in this study provides valuable insights for potential clinical applications. However, because of the bidirectional stoichiometric effect of PBM, the corresponding laser parameters must be selected for different inflammatory conditions to maximize efficacy and minimize potential side effects [17].

## Supplementary Information

Below is the link to the electronic supplementary material.

Supplementary Material 1 (DOCX 1.23 MB)

## Data Availability

The datasets generated and analyzed in this study are available from the corresponding author upon reasonable request. Supplementary information and additional raw data supporting the conclusions of this study will also be made available to qualified researchers without undue reservation. Researchers interested in accessing these datasets are encouraged to contact the corresponding authors.
