# Supplementary material for: Repeated photobiological regulation therapy alleviates inflammation in mice with experimental acute pancreatitis through ROS/NF-κB pathway
Source: Lasers Med Sci. 2026 Apr 7;41(1):66. doi: 10.1007/s10103-026-04868-7 (PMC13056793; doi:10.1007/s10103-026-04868-7)
Supplement: Supplementary file 1 — Supplementary Material 1 (DOCX 1.23 MB) [file 10103_2026_4868_MOESM1_ESM.docx]

**WB**

The original Western blot image is shown below; the experiment was performed in triplicate.


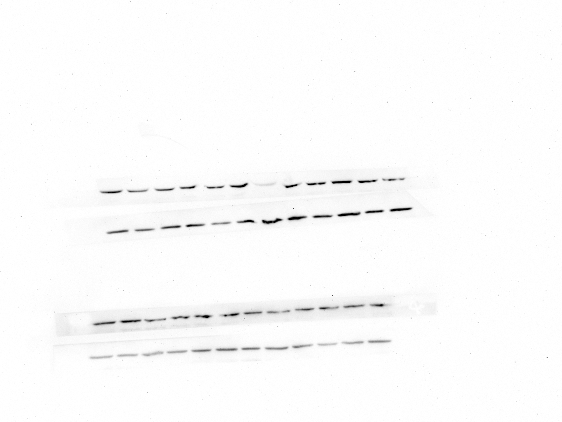


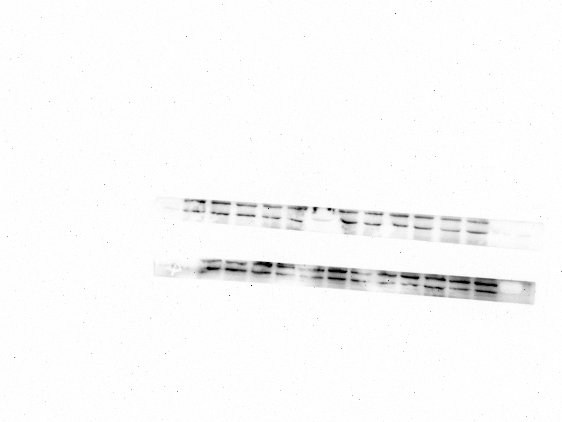


Phospho-NF-κB p65

β-actin

NF-κB p65

Phospho-NF-κB p65

NF-κB p65

β-actin


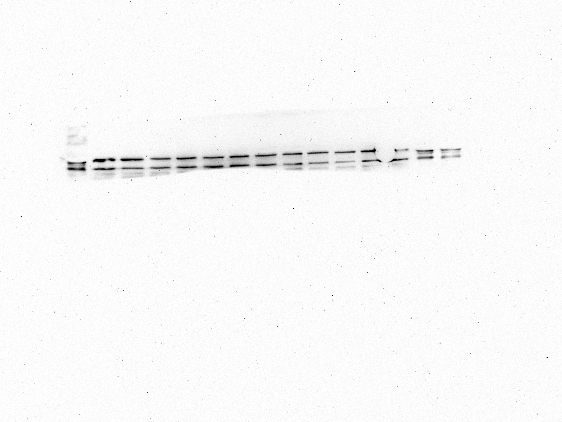

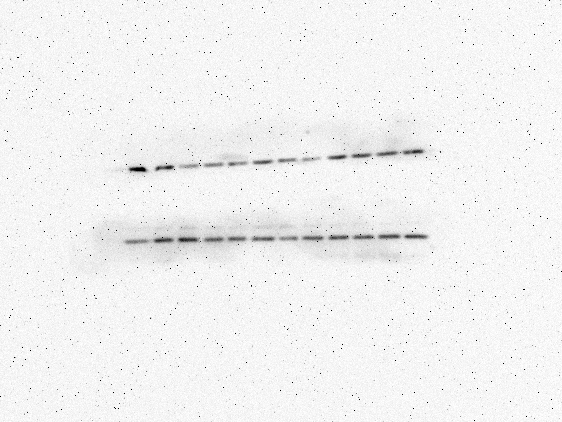

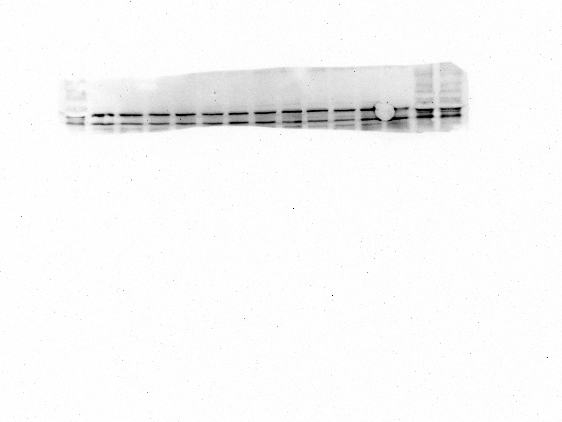

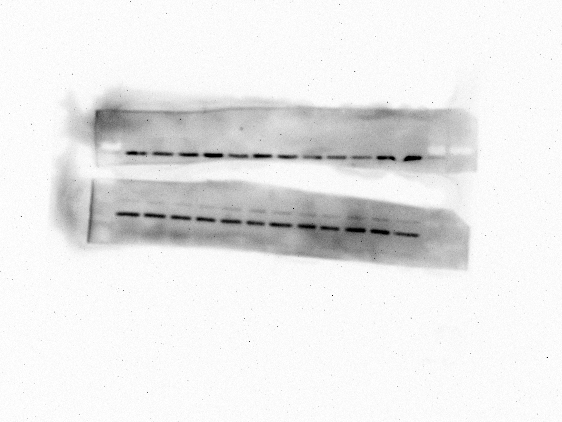


On the left side of the red line is the high-exposure Western Blot (WB) result image, where three markers are visible: two on the left and one on the right. On the right side of the red line is the low-exposure WB result image, so the positions of the markers cannot be displayed. The results presented in the article are those from the image on the right side of the red line.

NF-κB p65

NF-κB p65

Phospho-NF-κB p65

Phospho-NF-κB p65

β-actin

β-actin
